# Polyvinylalcohol-carbazate mitigates acute lung injury caused by hydrochloric acid

**DOI:** 10.3389/fphar.2024.1503648

**Published:** 2024-11-21

**Authors:** Caijuan Dong, Jielu Liu, Alessandro Quaranta, Xu Jing, Mu Nie, Craig E. Wheelock, Benjamin Murrell, Jonathan M. Coquet, Tim Melander Bowden, Thomas Engstrand, Mikael Adner

**Affiliations:** ^1^ Experimental Asthma and Allergy Research Unit, Institute of Environmental Medicine (IMM), Karolinska Institutet, Stockholm, Sweden; ^2^ Department of Cardiology, The First Affiliated Hospital of Soochow University, Suzhou, Jiang Su, China; ^3^ Unit of Integrative Metabolomics, Institute of Environmental Medicine (IMM), Karolinska Institutet, Stockholm, Sweden; ^4^ Department of Microbiology, Tumor and Cell Biology, Karolinska Institutet, Stockholm, Sweden; ^5^ Department of Respiratory Medicine and Allergy, Karolinska University Hospital, Stockholm, Sweden; ^6^ Department of Chemistry-Ångström Laboratory, Uppsala University, Uppsala, Sweden; ^7^ Department of Molecular Medicine and Surgery, plastic surgery section, Karolinska University Hospital, Stockholm, Sweden

**Keywords:** acute respiratory distress syndrome, aspiration pneumonia, oxidative stress, pharmacological treatment, intranasal administration

## Abstract

**Background:**

Acute lung injury (ALI) and acute respiratory distress syndrome (ARDS) are important causes of morbidity and mortality in critically ill patients. Gastric contents aspiration is one of the most common causes of ALI/ARDS. To date, there are still no specific and effective pharmacological treatments for ALI/ARDS. Polyvinylalcohol-carbazate (PVAC), a polymer that can bind endogenous aldehydes, neutralize oxidative stress and inhibit inflammatory factors, may be a potential treatment for ALI/ARDS.

**Methods:**

A hydrochloric acid (HCl) induced mouse model was employed to assess the effect of PVAC. The changes of lung mechanics, pulmonary edema, histology and immune cells, cytokines, and lipid mediators in bronchioalveolar lavage fluid (BALF) were investigated in HCl-challenged mice.

**Results:**

In the HCl model, PVAC administration alleviated airway hyperresponsiveness and improved pulmonary edema and damage. In addition, it decreased the recruitment of neutrophils to the lung, and inhibited the increase of IL-6, TNF-α and leukotriene B_4_.

**Conclusion:**

These data indicates that PVAC is a potential candidate for the treatment of ALI/ARDS induced by aspiration of gastric acid or for the control of “asthma-like” symptoms in patients with gastroesophageal reflux.

## 1 Introduction

Acute lung injury (ALI) and its more severe form, acute respiratory distress syndrome (ARDS), are common causes of morbidity and mortality in critically ill patients. The incidence of ARDS ranges from 4 to 75 cases per 100,000 people per year and the mortality from 40% to 50% (Villar et al., 2016). The main pathophysiological mechanisms of ALI/ARDS include increased vascular permeability, cytokine overproduction, leukocyte recruitment, and surfactant dysfunction (Meyer et al., 2021). At present, there is no specific drug for ARDS, so it is of great significance to develop more potential drugs for the treatment of ALI/ARDS.

Gastric contents aspiration is one of the most common causes of ALI/ARDS (Kaku et al., 2020). Aspiration occurs silently but very common in critically ill patients (Metheny et al., 2006). Inhaled substances may include food particles, stomach acid/hydrochloric acid (HCl), blood, or bacteria. Among them, HCl has the greatest effect on lung injury. About one-third of patients with aspiration pneumonia will develop a more severe and prolonged course associated with ALI/ARDS (Raghavendran et al., 2011). The pathogenic mechanisms of HCl-induced ALI/ARDS has shown to relate to reduced levels of anti-oxidating enzymes along with increased levels of lipid peroxidation in lung tissues indicating enhanced oxidative stress (El-Shahat et al., 2021), However, there are no effective pharmacological treatments for ALI/ARDS.

**FIGURE 1:**
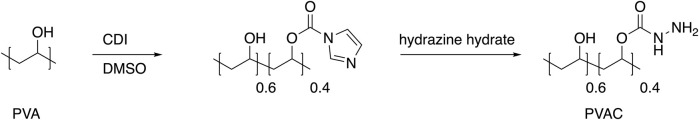
Synthesis of PVAC. PVAC is synthesized starting from polyvinyl alcohol (PVA). Activation of the hydroxyl group with carbonyl diimidazole (CDI) in DMSO renders a carbamate intermediate that upon treatment with hydrazine hydrate gives the product polyvinylalcohol-carbazate (PVAC). The degree of substitution of carbazate groups is 0.4.

Polyvinylalcohol-carbazate (PVAC) is a highly soluble polymer in aqueous solution (Figure 1), known for its capacity to bind endogenous aldehydes and neutralize oxidative stress (Sellberg et al., 2019). Studies have demonstrated that PVAC gel inhibits inflammation in osteoarthritis joints by quenching reactive oxygen species and lipid peroxidation products such as 4-hydroxynonenal (Fredriksson et al., 2017). Given these properties, it is of interest for investigating whether PVAC can inhibit ALI triggered by oxidative stress. Therefore, our study aimed to evaluate the efficacy of intranasal administration of PVAC in ALI/ARDS animal models induced by HCl.

## 2 Materials and methods

### 2.1 Ethical permission

All animal experiments were approved by the Stockholm ethics committee (the permit number:10,712-2020). Animals were housed at Astrid Fagraeus laboratory (KM-F) with 12-h dark/light cycle and had free access to food and water. All the *in vivo* experiments were performed at KM-F after at least 1 week of acclimatization.

### 2.2 HCl-induced ALI/ARDS mouse model and drug treatment

Male C57BL/6J mice (approximately 25 g, 8–12 weeks old) were ordered from Envigo (Horst, Netherlands). The mice were housed in a biosafety level 1 laboratory at Astrid Fagraeus Laboratory, Karolinska Institutet. Thirty minutes after intranasal administration of PVAC (3 mg/kg), the mice were instilled intratracheally with HCl (pH 1.5; 40 µL). Three hours post HCl exposure, the airway responses to methacholine were assessed followed by bronchoalveolar lavage and sample collections. The left lungs were fixed in 4% formaldehyde and right lungs were snap-frozen for further studies.

The dose rationale for PVAC is based on safety parameters, including molecular behavior and toxicological outcomes (unpublished and proprietary data with permission by T. Bowden and T. Engstrand, PVAC Medical Technologies LTD.). Higher concentrations of PVAC, a polymer, can increase viscosity and cause aggregation. *In vitro* and preclinical studies set the upper concentration limit, with the current study using 3 mg/mL, which is below this limit. Safety studies showed that both rats and rabbits tolerated lower doses of PVAC, but higher doses led to granular material deposition in phagocytic cells. The current study used a dose below the no-observed-adverse-effect-level (NOAEL), with no such depositions observed in the lungs of treated animals.

### 2.3 Airway responsiveness test

Mice were anesthetized with ketamine hydrochloride (75 mg/kg, Ketaminol^®^ Vet., Intervet, Stockholm, Sweden) and medetomidine hydrochloride (1 mg/kg, Cepetor®Vet., VETMEDIC, Stockholm, Sweden). An 18-gauge blunt metal cannula was inserted into the trachea and secured in place with a nylon suture. Animals were placed on a heating pad and connected to the flexiVent system (flexiVent FX4, SCIREQ Inc., Montreal, Qc, Canada), where airway responses to methacholine (MCh) (1.56 mg/mL to 12.5 mg/mL) were assessed.

### 2.4 Wet to dry weight ratio

The left lungs were harvested and weighed to obtain the wet weight. The lungs were then placed into a drying oven at 80°C for 24 h to obtain the dry weight. The wet to dry weight ratio was calculated to evaluate the degree of lung edema (Wet to dry ratio = wet weight/dry weight).

### 2.5 Bronchoalveolar lavage

Bronchoalveolar lavage was performed directly after lung function measurements. 0.8 mL cold PBS was gently lavaged twice in the lung. The bronchoalveolar lavage fluid (BALF) was centrifuged at +4°C, 1,000 rpm, for 10 min and the supernatant was stored at −80°C until use. Total cell number was counted using Turk staining under microscope and expressed as cells·ml^−1^ BALF. Differential cell counts were performed on May-Grünwald/Giemsa stained cytospins, counting a minimum of 300 cells, in a blinded manner.

### 2.6 H&E histological staining

Hematoxylin and eosin (H&E) staining of paraffin-embedded lung tissue sections were performed to evaluate the lung damage. Lung tissues were fixed with 4% formalin for 24 h, embedded in paraffin, and sectioned at 5-μm thickness. After deparaffinization and dehydration, sections were stained with H&E and were photographed using a light microscope (Nikon Eclipse TS100) equipped with a camera (DS-Fi1; Nikon) and software (NIS-Elements F3.0). Lung injury was scored by assessing the degree of inflammatory cell infiltration, hemorrhage, interstitial and alveolar edema and thickness of the alveolar septum in five randomly selected fields under a light microscope from 0-4 (0: No damage, 1: Mild damage, 2: moderate damage, 3: severe damage and 4: highly severe histological damage).

### 2.7 ELISA

Enzyme-linked immunosorbent assay (ELISA) was used for determining the protein concentrations of mouse IL-6 (M6000B, R&D Systems), TNF-α (RAB0477, Sigma Aldrich), and Myeloperoxidase (MPO) (RAB0374, Sigma Aldrich). The ELISAs were performed according to the manufacturers’ protocols. Absorbance values were detected at 450 nm using a microplate reader and concentrations were calculated according to the standard curves plotted.

### 2.8 Lipid mediator characterizations

Lipid mediators were extracted and analyzed by following a previously published method (Kolmert et al., 2018). Briefly, 800 µL of BALF were added with an internal standard solution, diluted to 1.5 mL with a solution consisting of 0.2 M Na_2_HPO_4_/0.1 M citric acid (58/42 v/v, pH 5.6), and extracted on preconditioned ABN Evolute Express solid-phase extraction cartridges (3cc/60 mg, Biotage, Uppsala, Sweden). A total of 110 lipid mediators were quantified by liquid chromatography coupled to tandem mass spectrometry (LC-MS/MS) on an Acquity UPLC coupled to a Xevo TQ-XS mass spectrometer (Waters, Milford, MA, USA).

### 2.9 Statistical analysis

The experimental results were analyzed using Graphpad Prism 9.0 statistical software (GraphPad, USA). Data were presented as mean ± SEM. Two-group data were analyzed by unpaired t-test, and multiple-group data were analyzed by one-way or two-way ANOVA. Sidak’s multiple comparisons test was used for pairwise comparisons in the analysis of variance. *P*-value <0.05 was considered statistically significant.

## 3 Results

### 3.1 PVAC improves lung function in HCl mice

To assess airway responsiveness, HCl-challenged mice were connected to the flexiVent system and exposed to increasing concentrations of methacholine (MCh) aerosol. Several parameters were recorded, including the total resistance (Rrs) and elastance (Ers) of the respiratory system, as well as the resistance in the conducting airways (Newtonian resistance; Rn), peripheral lung tissue damping (G), and peripheral tissue elastance (H).

**FIGURE 2:**
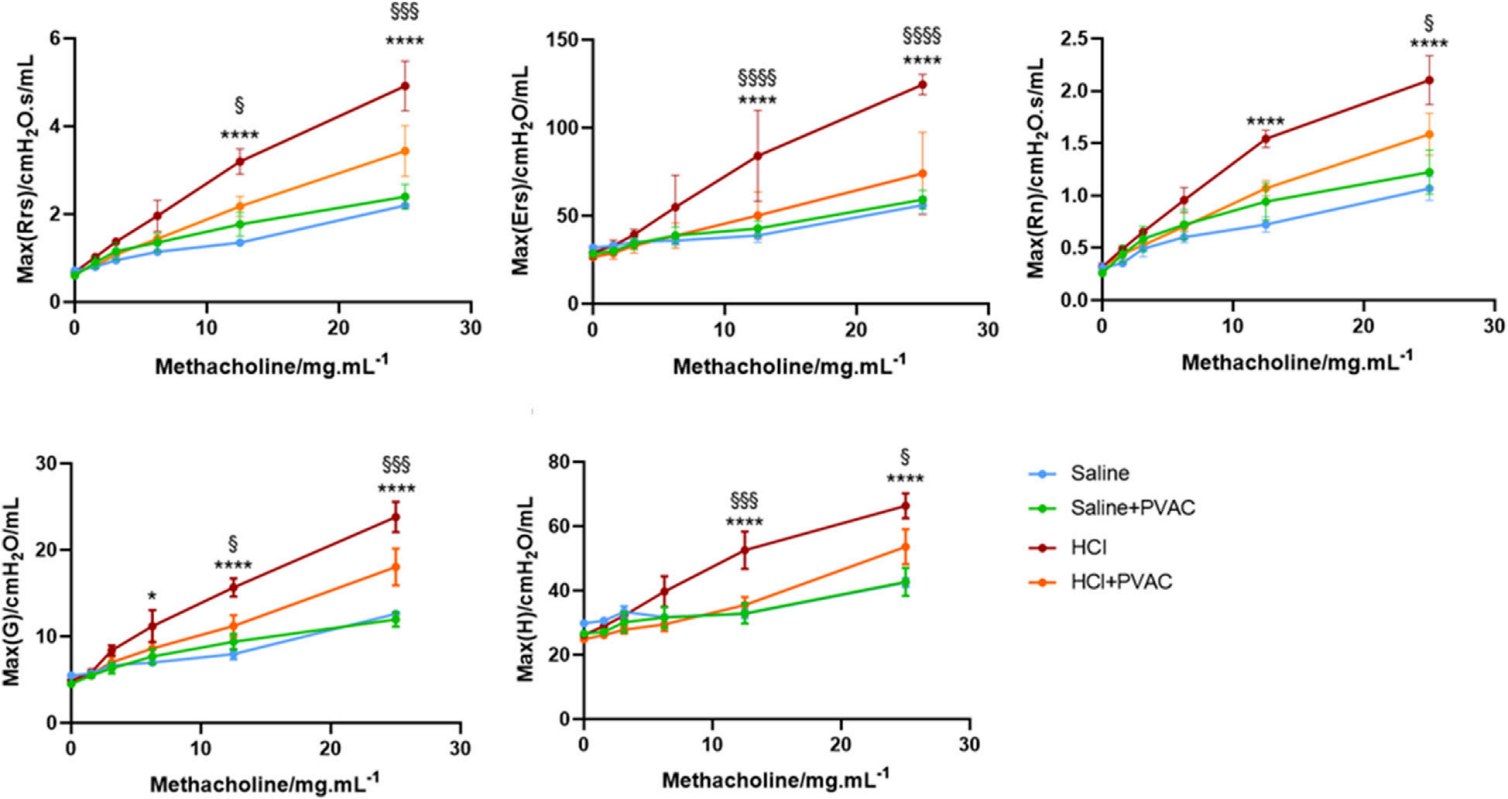
Airway responsiveness to Methacholine in HCl-induced ALI/ARDS mice. The AHR was measured with a small animal ventilator (flexiVent; Scireq), as previously described. Resistance of the respiratory system (Rrs), elastance of the respiratory system (Ers), Newtonian Resistance (Rn), tissue damping (G) and tissue elastance (H) were recorded. The results were expressed as mean ± SEM (n = 4–5). saline: animals exposed to saline and received saline pretreatment. saline + PVAC: animals exposed to saline and received PVAC pretreatment. HCl: animals exposed to HCl and received saline pretreatment. HCl + PVAC: animals exposed to HCl and received PVAC pretreatment. *P< 0.05, ****P< 0.0001 vs respective saline, §*P*< 0.05, §§§*P*< 0.001, and §§§§*P*< 0.0001 vs respective HCl + PVAC.

When comparing the saline group and the saline + PVAC group, no significant differences were found in Rrs, Ers, Rn, G, and H (*p* > 0.05) (Figure 2), whereas HCl-challenged caused an increase of these parameters at 6.25 and 12.5 mg/mL MCh (*p* < 0.05). Pretreatment with PVAC dampened the increase induced by HCl in all parameters (*p* < 0.05).

### 3.2 PVAC improves lung edema and lung damage in HCl mice

**FIGURE 3:**
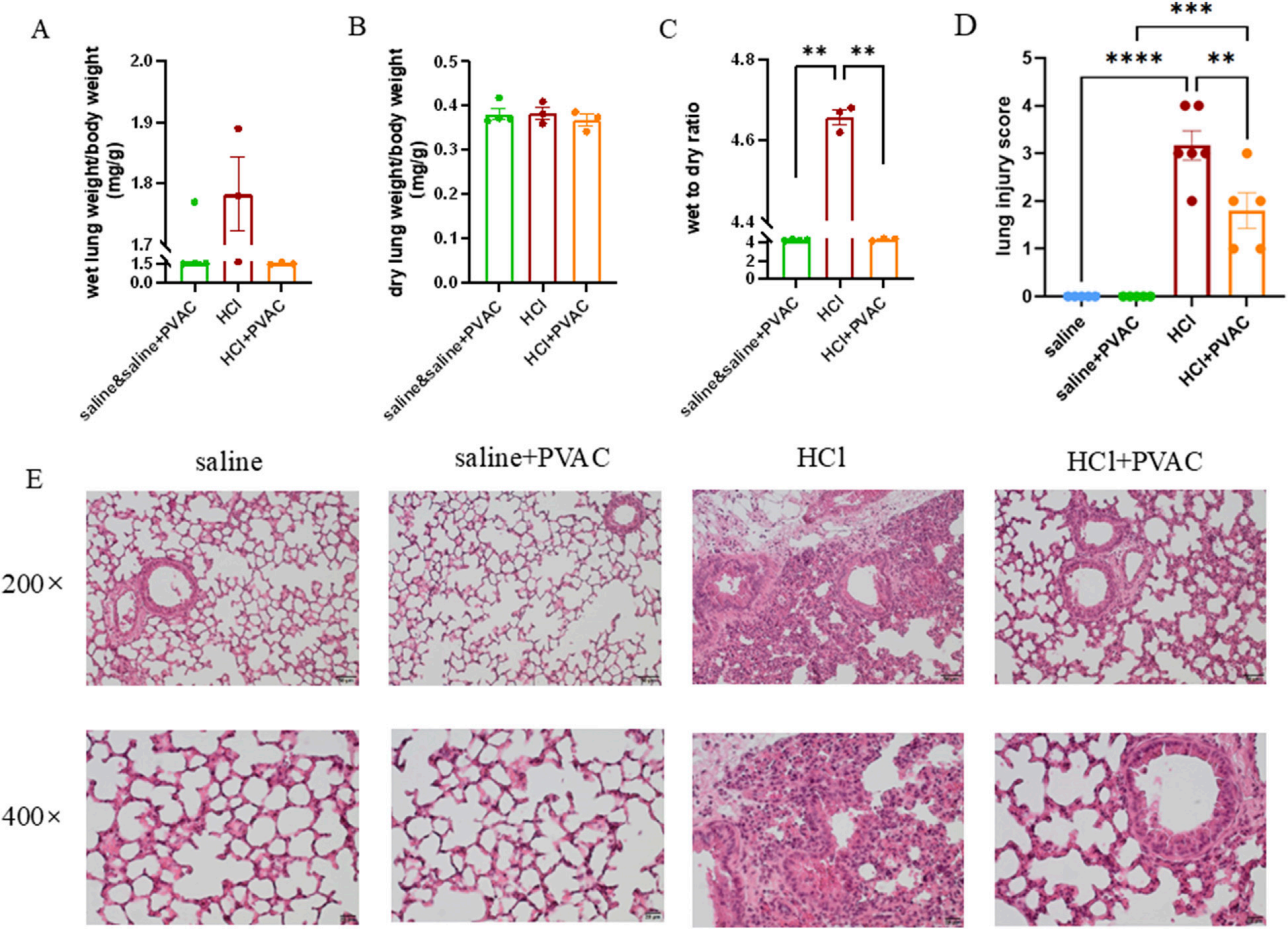
HCl-induced lung edema and histological changes in lung tissues. Left lungs were harvested. Some of them were weighed and dried in 80°C oven for 24h to obtain the dry weight. Wet to dry weight ratio was calculated as wet weight/dry weight. The other left lungs were fixed, embedded in paraffin and cut into 5 μm slices. After H&E staining, histological examination was performed by light microscopy and assessed based on the lung injury score. **(A)** Wet weight of left lung (n = 3-4). **(B)** Dry weight of left lung (n = 3-4). **(C)** Wet to dry weight ratio of left lung (n = 3-4). **(D)** lung injuty score (n = 5-6). **(E)** Representative images of H&E-stained lung sections from HCl-challenged mice with and without PVAC pretreatment (bar scale: 50μm and 20 μm). The results were expressed as mean ± SEM. saline: animals exposed to saline and received saline pretreatment. saline + PVAC: animals exposed to saline and received PVAC pretreatment. HCl: animals exposed to HCl and received saline pretreatment. HCl + PVAC: animals exposed to HCl and received PVAC pretreatment. **P< 0.01, ***P< 0.001, and ****p < 0 .0001.

Examining whether PVAC affected pulmonary edema, the calculation of the wet to dry weight ratio of the left lung tissue showed that HCl exposure significantly had a significantly higher ratio than the control group *(P*< 0.01), whereas PVAC pretreatment significantly reduced HCl-induced pulmonary edema (*P*< 0.01) (Figures 3A–C).

Lung damage was assessed using H&E-stained slides. Compared to control mice (saline), no histological changes were observed in the lung specimens of saline and PVAC-treated mice (Figure 3D). However, HCl exposure induced massive neutrophil infiltration around the pulmonary vascular and interstitial spaces, marked swelling of the alveolar walls, severe hemorrhage, and significant damage to the alveolar structure (Figure 3E). The lung injury score of the HCl + PVAC group was significantly lower than that of the HCl group (*p* < 0.01), but still higher than that of the control group (*p* < 0.001) (Figure 3D).

### 3.3 PVAC alleviates inflammation in BALF from HCl mice

**FIGURE 4:**
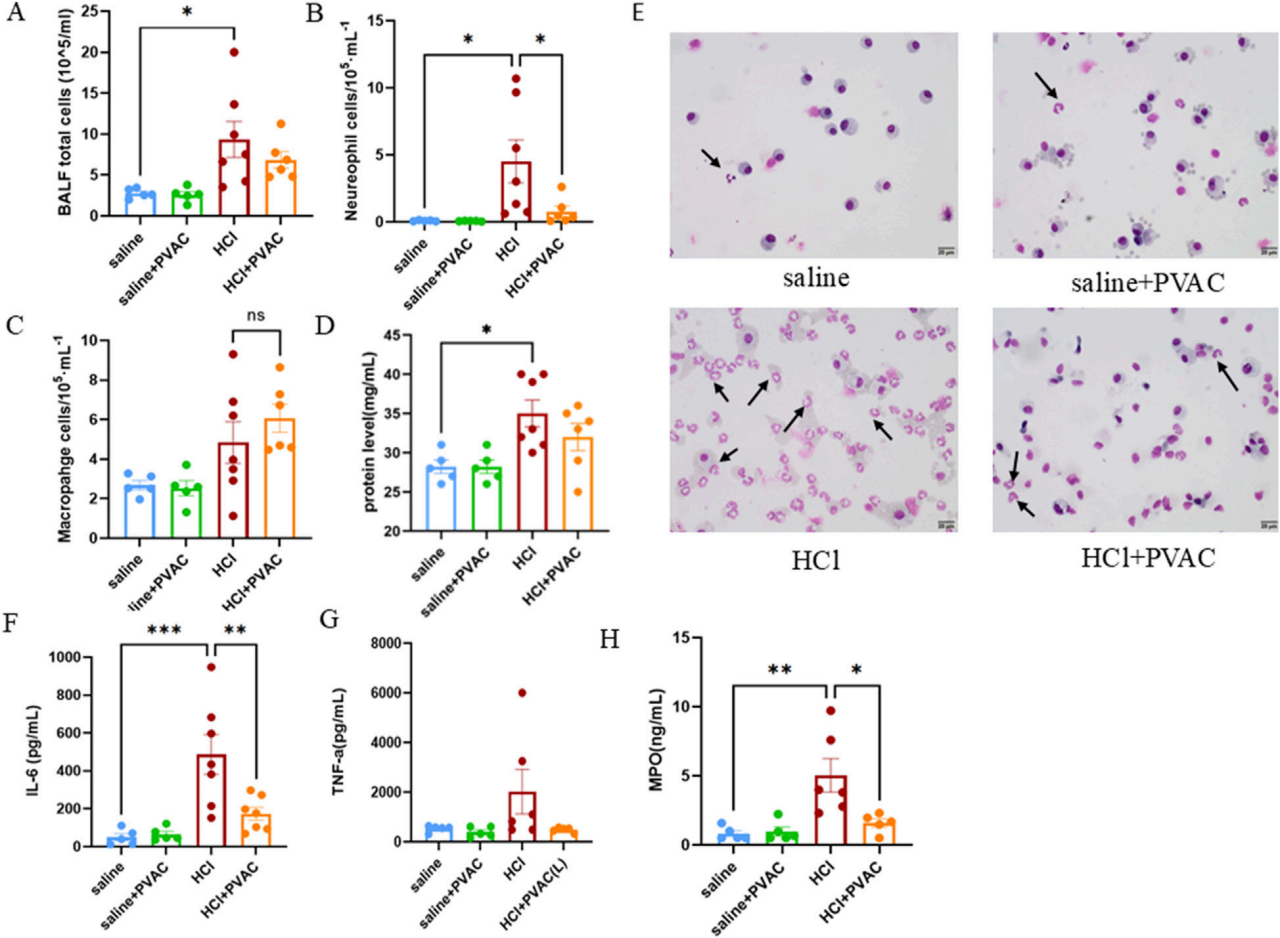
Leukocytes, total protein levels and cytokine levels measured in the BALF of HCl-induced ALI/ARDS mice. **(A)** The number of total cells in the BALF. **(B)** The number of neutrophils in the BALF. **(C)** The number of macrophages in the BALF. **(D)** Total protein levels in the BALF. **(E)** Representative images of May-Grünwald/Giemsa-stained cytospin slides from HCl-challenged mice with and without PVAC pretreatment (bar scale: 20 μm). **(F)** IL-6 levels in the BALF. **(G)** TNF-a level in the BALF. **(H)** MPO level in the BALF. The results were expressed as mean ± SEM(n = 5-7). saline: animals exposed to saline and received saline pretreatment. saline + PVAC: animals exposed to saline and received PVAC pretreatment. HCl: animals exposed to HCl and received saline pretreatment. HCl + PVAC: animals exposed to HCl and received PVAC pretreatment. *P< 0.05, **P< 0.01, and ***P< 0.001.

To examine inflammation, an analysis of inflammatory cells in bronchoalveolar lavage fluid (BALF) was conducted. Exposure to HCl significantly increased the total number of cells, neutrophils, and macrophages compared to the saline group (*p* < 0.05) (Figures 4A–C). PVAC treatment significantly reduced the number of neutrophils in the BALF of HCl-challenged mice (*p* < 0.05) (Figure 4B), while the total number of cells and macrophages were not significantly altered. Moreover, HCl significantly increased the total protein levels in BALF (Figure 4D), suggesting that HCl could cause vascular leakage. However, there was no significant change in protein content in BALF in the HCl + PVAC group compared to the HCl group.

As neutrophils is one major important leukocyte in ALI/ARDS (Meyer et al., 2021), the levels of IL-6 and TNF-α associated with neutrophil infiltration and vascular permeability, and MPO as an indicator of neutrophil infiltration were measured in the BALF. Compared with the control group, HCl significantly increased the level of IL-6 in BALF (Figure 4G), whereas TNF-α increased slightly but did not reach statistical significance (Figure 4H). PVAC pretreatment significantly decreased the level of IL-6 in BALF of HCl-challenged mice and the level of TNF-α was also decreased but did not reach statistical significance. For MPO, HCl treatment resulted in a significant increase in MPO activity in BALF which was inhibited by PVAC.

### 3.4 PVAC modulates lipid mediators in HCl mice

**FIGURE 5:**
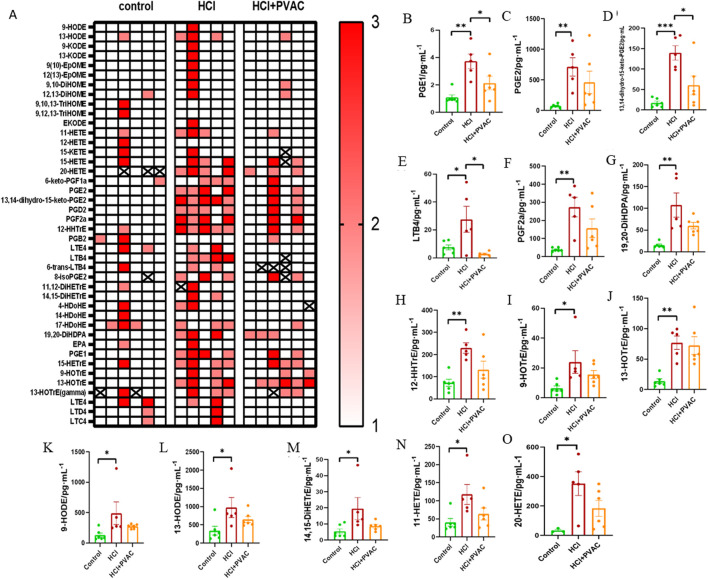
Lipid mediators measured in the BALF of HCl-induced ALI/ARDS mice. A broad of pro- and anti-inflammatory lipid mediators were detected using LC-MS/MS method. **(A)** Heat map. 1 (white) indicates concentrations are between the lowest and first tertile, 2 (light red) between the first and second tertile, and 3 (thick red) between the second tertile and maximum concentration; **(B)** PGE1, prostaglandin E1; **(C)** PGE2, prostaglandin E2; **(D)** 13,14-dihydro-15-keto-PGE2, 13,14-dihydro-15-keto prostaglandin E2; **(E)** LTB4, leukotriene B4; **(F)** PGF2α, prostaglandin F2α; **(G)** 19,20-DiHDPA, 19,20-dihydroxy-docosapentaenoic acid; **(H)** 12-HHTrE, 12-hydroxyheptadecatrienoic acid; **(I)** 9-HOTrE, 9-hydroxyoctadecatrienoic acid; **(J)** 13-HOTrE, 13-hydroxyoctadecatrienoic acid; **(K)** 9-HODE, 9-hydroxyoctadecadienoic acid; **(L)** 13-HODE, 13-hydroxyoctadecadienoic acid; **(M)** 14,15-DiHETrE, 14,15-dihydroxyeicosatrienoate; **(N)** 11) -HETE,11-hydroxyeicosatetraenoic acid; **(O)** 20-HETE, 20-hydroxyeicosatetraenoic acid. Results were presented as mean ± SEM (n = 5–6). control: saline and saline + PVAC, HCl: HCl treatment, HCl + PVAC: PVAC pretreatment + HCl treatment. Statistical comparisons between groups were performed using one-way ANOVA, *P< 0.05, **P< 0.01, and ***P< 0.001.

Bioactive lipid mediators are involved in a wide range of physiological and pathological processes, particularly in inflammatory responses (Stables and Gilroy, 2011; Serhan et al., 2014). To investigate the response of lipid mediators in BALF to HCl stimulation and the effect of PVAC on lipid mediators, a broad range of pro- and anti-inflammatory lipid mediators was quantified using LC-MS/MS. HCl treatment increased 42 out of 110 measured lipid mediators, of which 13 were significant. Compared with the control group, HCl caused a significant increase (*P*< 0.05) of prostaglandin (PG) E_1,_ PGE_2_, 13,14-dihydro-15-keto-PGE_2_, leukotriene B_4_ (LTB_4_), PGF_2α_, 19,20-dihydroxy-docosapentaenoic acid (19(20)-DiHDPA), 12-hydroxy-heptadecatrienoic acid (12-HHTrE), 9-hydroxy-octadecatrienoic acid (HOTrE), 13-HOTrE, 9- hydroxy-octadecadienoic acid (HODE), 13-HODE, 14,15-dihydroxy-eicosatrienoic acid (14,15-DiHETrE), 11-hydroxy-eicosatetraenoic acid (HETE), 20-HETE. Compared with HCl, PVAC could significantly reduce PGE_1_ (*P*<0.05), 13,14-dihydro-15-keto-PGE_2_ (*P*< 0.05) and LTB4 (*P*< 0.05) (Figures 5B, D, E).

## 4 Discussion

To our knowledge, this is the first study to evaluate the effect for airway exposure of PVAC. This was assessed on ALI/ARDS animal models. The study revealed the prophylactic effect of PVAC in HCl-induced ALI/ARDS mice. No adverse effects were observed in C57BL/6J mice treated with PVAC, suggesting the safety of PVAC in respiratory diseases. Moreover, pre-treatment of PVAC significantly (i) reduced HCl-induced airway hyperresponsiveness and (ii) improved HCl-induced pulmonary edema and lung histopathological damage. These effects may be achieved by inhibiting pro-inflammatory cytokines such as IL-6 and TNF-α, and lipid mediators such as LTB_4_, thereby reducing neutrophil infiltration and vascular permeability.

Airway hyperresponsiveness is defined as an exaggerated and accelerated constriction of the airway to various stimuli that does not elicit a comparable response in healthy subjects. Consistent with Gilman and colleagues’ findings, inhalation of HCl was observed to induce airway hyperresponsiveness in mice (Allen et al., 1985). In the clinic, studies have shown that patients with gastroesophageal reflux suffer airway hyperresponsiveness and “asthma-like” symptoms (Paoletti et al., 2021). There are several possible mechanisms for this such as 1) the barrier disruption caused by HCl facilitates the egress of MCh from the airway lumen to the underlying smooth muscle, resulting in airway hyperresponsiveness, 2) edema within and around the airways that reduces the airway lumen, and 3) elevated cytokines caused by HCl, such as TNF-α, induce calcium influx into airway smooth muscle cells and lead to smooth muscle contraction (Spond et al., 2004). Our study has demonstrated that PVAC can reduce airway hyperresponsiveness in HCl-challenged mice, and a possible mechanism is that PVAC reduces the airway smooth muscle contraction by inhibiting the calcium influx mediated by cytokines such as TNF-α. Moreover, it is possible that PVAC could have important clinical implications for patients with gastroesophageal reflux.

Infiltration and accumulation of leukocytes, especially neutrophils and macrophages in the interstitial and alveolar spaces of the lung, is one of the most important pathological hallmarks of ALI/ARDS (Matthay et al., 2012; Abraham, 2003). Although rapid and appropriate influx of neutrophils from the circulation into the lung is essential for clearance of microbial pathogens and debris from the alveolar space, excessive and persistent sequestration of neutrophils may cause additional damage to the lungs, by releasing several toxic mediators, including proinflammatory cytokines and procoagulant molecules, thereby exacerbating ALI (Abraham, 2003; Bhattacharya and Matthay, 2013). It has been reported that neutrophils in the BALF of ARDS patients is closely related to disease severity and poor prognosis (Abraham, 2003). In our study, a significant enhanced neutrophil and macrophage infiltration in BALF were observed in HCl mice. No effects on eosinophils were found suggesting that the effect mainly is T_H_1-driven. PVAC pretreatment significantly attenuated HCl-induced neutrophil influx into the lungs without an effect on the macrophages. This selective effect on neutrophils may be due to inhibition of LTB_4_ (discussed below). The HCl-induced increase of MPO also indicates neutrophil engagement, a result further corroborated by the results that PVAC also inhibited MPO activity. Edema and protein extravasation are considered indicators of vascular leakage. The wet to dry ratio of left lung tissue and total protein content in the BALF were measured to evaluate the degree of edema and protein leakage. The significantly elevated wet-dry ratio of left lung tissue and total protein content in the BALF were observed in HCl-challenged mice, suggesting HCl increases the pulmonary capillary permeability. However, it can be alleviated by PVAC. These results suggest that PVAC attenuated neutrophil influx and vascular leakage in HCl-challenged mice, thereby ameliorating lung pathology.

IL-6, as a multifunctional cytokine, plays an important role in acute inflammatory responses. Sustained elevation of IL-6 in plasma and BALF in ARDS patients has been shown to be inversely associated with disease outcome and patient survival (Meduri et al., 1995; Casey et al., 1993). Christian et al. confirms that IL-6 mediates neutrophil infiltration and pulmonary edema (Hierholzer et al., 1998). Similar to IL-6, TNF-α, mainly secreted by macrophages, is also involved in the acute inflammatory responses. *In vivo* and *in vitro* experiments have demonstrated that TNF-α increases alveolar capillary wall permeability under pathological conditions (Stephens et al., 1988), causing pulmonary edema. Therefore, we assessed the levels of IL-6 and TNF-α in BALF. It was found that IL-6 was significantly increased in the BALF of HCl mice, and TNF- α was also increased but did not reach statistical significance. Speculating, this result may indicate that TNF-α is involved, which also has been shown previously in a similar model of HCl-induced lung injury (Bastarache and Blackwell, 2009). Decreased IL-6 and TNF-α in BALF were observed in PVAC-treated, HCl-challenged mice. Therefore, PVAC may reduce HCl-induced vascular leakage and neutrophil infiltration by inhibiting IL-6 and TNF-α signaling axis, thereby improving pulmonary edema and lung pathological damage. IL-6 and TNF-α have been confirmed to be critical cytokines in the pathogenesis of COVID-19 (Ye et al., 2020), and their levels are correlated with the severity and prognosis of COVID-19. Inhibition of “cytokine storm” has been recognized as an important strategy for the treatment of severe COVID-19. Therefore, PVAC is a potential candidate for the SARS-CoV-2 treatment.

LTB_4_ is known to be one of the most potent neutrophil chemotactic agents and plays an important role in host defense against infection by interacting with its high-affinity receptor BLT1 (He et al., 2020). Our study found that HCl caused a significant increase of LTB_4_ levels in the BALF, which further corroborated by the results that HCl induced neutrophils influx. PVAC was observed to significantly reduce LTB_4_ levels in the BALF of HCl-challenged mice. As PVAC is a potent nucleophilic polymer it can interact with electrophilic molecules (Liu, 2013). The PVAC mode of action is most likely the interaction and inhibition of electrophilic entities in the inflammatory cascade such as hydrogen peroxide, aldehydes and LTA_4_ (a LTB_4_ precursor). Therefore, PVAC possibly ameliorated lung injury by inhibiting the LTB_4_-driven neutrophil infiltration in the lungs of HCl mice. Targeted lipidomic analysis of BALF was performed by LC-MS/MS in 25 healthy controls and 33 COVID-19 patients requiring mechanical ventilation. The study found that fatty acids and inflammatory lipid mediators were increased in the BALF of severe COVID-19 patients. Thromboxane, prostaglandins, leukotrienes (especially LTB_4_ and LTE_4_) were significantly increased. Monohydroxylated 15-lipoxygenase metabolites from linoleic, arachidonic, eicosapentaenoic, and docosahexaenoic acids were increased, too. This indicates that the “lipid mediator storm” that occurs in severe COVID-19 involves both pro- and anti-inflammatory lipids (Archambault et al., 2021). Therefore, PVAC is expected to improve the condition of COVID-19 patients by inhibiting the “lipid mediator storm”.

This study also has limitations. 1) Human aspiration of gastric fluid is not simply inhalation of HCl, but rather the more complex gastric contents, which include particulate matter, bacterial products, and suspensions of cytokines. Thus, whether PVAC can be used for the treatment of ALI/ARDS caused by acidic substances in humans remains to be further studied. 2) Only the preventive effects of PVAC were investigated. These experiments are the first to study the effect of PVAC on inflammatory challenge in the lung. However, the marked effect of PVAC indicates a potential for promising future studies studying the effect during different time points and therapeutic properties. 3) The mechanisms of action for PVAC are not fully understood. Investigations into how the effect of PVAC is related to oxidative stress will be investigated in future studies.

## 5 Conclusion

This study shows that administration of PVAC may improve airway hyperresponsiveness, pulmonary edema and pulmonary pathological changes in HCl-induced ALI/ARDS model by reducing the levels of pro-inflammatory cytokines, lipid mediators, and the accumulation of neutrophils in the lungs, making PVAC a potential candidate for the treatment of ALI/ARDS induced by aspiration of gastric acid or for the control of “asthma-like” symptoms in patients with gastroesophageal reflux.

## Data Availability

The raw data supporting the conclusions of this article will be made available by the authors, without undue reservation.
